# Physiological Roles of Plant Methionine Sulfoxide Reductases in Redox Homeostasis and Signaling

**DOI:** 10.3390/antiox7090114

**Published:** 2018-08-29

**Authors:** Pascal Rey, Lionel Tarrago

**Affiliations:** 1 Laboratoire d’Ecophysiologie Moléculaire des Plantes, Aix Marseille University, CEA, CNRS, BIAM, F-13108 Saint Paul-Lez-Durance, France; 2 Laboratoire de Bioénergétique Cellulaire, Aix Marseille University, CEA, CNRS, BIAM, F-13108 Saint Paul-Lez-Durance, France; lioneltarrago@msn.com

**Keywords:** methionine, methionine sulfoxide, methionine sulfoxide reductase, physiological function, protein, plant, repair, redox homeostasis, signaling, stress

## Abstract

Oxidation of methionine (Met) leads to the formation of two *S*- and *R*-diastereoisomers of Met sulfoxide (MetO) that are reduced back to Met by methionine sulfoxide reductases (MSRs), A and B, respectively. Here, we review the current knowledge about the physiological functions of plant MSRs in relation with subcellular and tissue distribution, expression patterns, mutant phenotypes, and possible targets. The data gained from modified lines of plant models and crop species indicate that MSRs play protective roles upon abiotic and biotic environmental constraints. They also participate in the control of the ageing process, as shown in seeds subjected to adverse conditions. Significant advances were achieved towards understanding how MSRs could fulfil these functions via the identification of partners among Met-rich or MetO-containing proteins, notably by using redox proteomic approaches. In addition to a global protective role against oxidative damage in proteins, plant MSRs could specifically preserve the activity of stress responsive effectors such as glutathione-*S*-transferases and chaperones. Moreover, several lines of evidence indicate that MSRs fulfil key signaling roles via interplays with Ca^2+^- and phosphorylation-dependent cascades, thus transmitting ROS-related information in transduction pathways.

## 1. Introduction

Post-translational modifications (PTMs) are covalent modifications that occur after protein synthesis, and change the chemical repertoire of amino acids by altering or introducing functional groups. Thereby, PTMs fulfill critical roles in the control of protein conformation, enzyme activity, or subcellular localization. Proteins are subject to numerous types of PTMs such as phosphorylation, glycosylation, acetylation or hydroxylation, which consist generally in enzymatic modifications of amino acid lateral chains. Special cases of PTMs are “redox PTMs”, in which amino acids are modified by reductive or oxidative reactions. The most current redox PTMs are oxidative modifications due to reaction with reactive oxygen species (ROS). As these species are metabolically produced, redox PTMs are tightly linked to changes in the global cell redox homeostasis such as oxidative stress, but also to more subtle variations that are timely and spatially controlled at the subcellular level. All amino acids are subject to oxidative modifications in their lateral chain that are generally irreversible [1]. This is the case for example of carbonylation, the level of which is considered as a marker of oxidative damage [2]. Interestingly, sulfur-containing residues are highly prone to oxidative PTMs that are reversible in many cases. Cysteine (Cys) undergoes modifications such as disulfide bridge formation or *S*-glutathionylation that are reversed through the action of oxidoreductases belonging to the thioredoxin (TRX) and glutaredoxin (GRX) families [3]. TRXs are ubiquitous, small disulfide reductases carrying two catalytic cysteines in a Cys-Xxx-Xxx-Cys active site signature. They form complex families in plants, likely indicating functional specialization depending on subcellular localization and expression pattern [4]. TRXs get reducing power from two main sources: the reduced form of nicotinamide adenine dinucleotide phosphate (NADPH) and the photosynthetic electron transfer chain. GRXs, which are closely related to TRXs, generally use glutathione as an electron donor [5,6], and form large families in higher plants [7]. TRXs and GRXs control multiple metabolic reactions, developmental processes, and stress responses by modulating the redox status of Cys in a very broad range of proteins [4,8,9,10]. This is evidenced by the number and diversity of interacting partners in photosynthetic organisms [11,12,13]. Among them, methionine sulfoxide reductases (MSRs), which control the redox status of Met, are well characterized.

**Figure 1:**
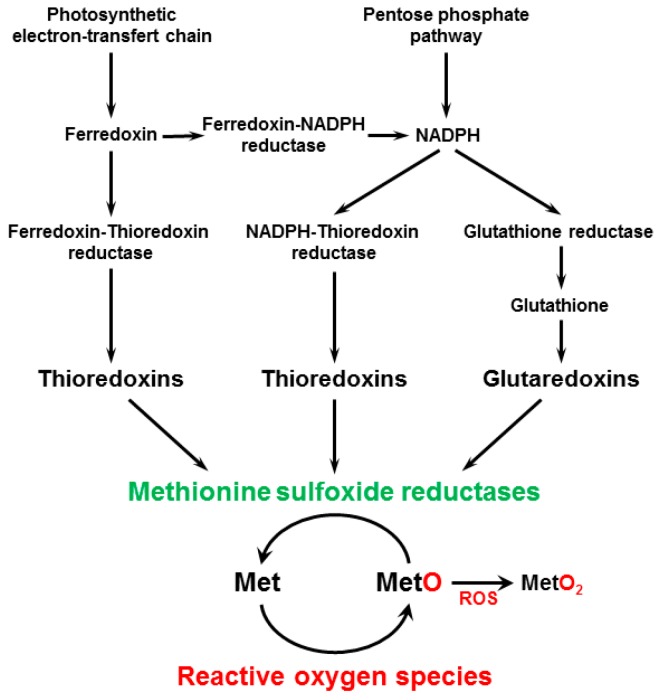
Reducing pathways of plant methionine sulfoxide reductases (MSRs). Methionine (Met) can be oxidized into Met sulfoxide and met sulfone, the latter modification being irreversible. Two main paths supply electrons to MSRs that allow Met regeneration from Met sulfoxide (MetO): on one hand, the path from photosynthetic electron chain to thioredoxins that takes place in chloroplast, and on the other hand, the one from reduced nicotinamide adenine dinucleotide phosphate (NADPH) to thioredoxin that is localized in cytosol. Other routes, involving notably glutaredoxins, participate in the reduction of atypical MSRs harboring for example one unique catalytic cysteine, in chloroplast. ROS: reactive oxygen species.

The oxidation of free and peptide-bound Met, the other sulfur-containing amino acid, results in the formation of Met sulfoxide (MetO) and Met sulfone (MetO_2_), the latter modification being irreversible (Figure 1). Quantitative estimations indicate that MetO can range from low micromolar concentration in basal condition to low millimolar concentration during acute oxidative stress [14], corresponding to up to 40% of total Met [15]. MetO is reduced back to Met by MSRs [16]. Met oxidation leads to the formation of *S*- and *R*-MetO diastereoisomers, and two types of MSRs, A and B, specifically reduce the *S*- and *R*-MetO isomers, respectively. Although displaying a very close biochemical function, they do not share any sequence similarity, but have a mirrored active site designed to accommodate each MetO diastereoisomer [16,17,18,19]. Further, whereas MSRAs generally reduce both the peptide-bound MetO and the free form with similar efficiency, MSRBs are generally more efficient in reducing the peptide-bound form [20], except for some isoforms in plants [21]. Most MSRs harbor two redox-active Cys and function using a three-step catalytic mechanism involving the formation of a Cys sulfenic acid intermediate, the subsequent formation of a disulfide bond and the regeneration of activity, most often by a TRX reducing system [18,22,23,24] (Figure 1). Mechanisms involving GRXs, unusual TRX types or other thiol-compounds allow the regeneration of atypical MSR proteins carrying only one catalytic Cys [25,26,27,28,29,30,31]. Note that some plant MSRA isoforms do not harbor a catalytic Cys residue [32], raising the question of their biochemical function.

In bacterial, yeast, and mammal cells, MSRs fulfill essential functions in stress tolerance and during ageing. For instance, in yeast, deletion and overexpression of *MSRA* results in reduced and increased viability, respectively [33], and the abundance of MSRs decreases upon ageing and diseases in mammal cells [34,35]. Modifying the expression of *MSRA* genes revealed their participation in the responses to oxidative stress generated by hydrogen peroxide (H_2_O_2_) or methyl viologen (MV), which generates superoxide [36,37]. Based on these data, a direct antioxidant function was first attributed to these enzymes in elimination of ROS via cyclic oxidation of Met in proteins and reduction by MSRs [15,38]. Additionally, many evidence in various organisms revealed that the control of Met redox status is a key step in signaling pathways. For instance in *E. coli*, exposure to HOCl leads to Met oxidation in the hypochlorite-responsive transcription factor (HypT) and subsequent activation of the expression of genes participating in protective mechanisms against this toxic compound [39]. In animals, the reversible oxidation of Met by ROS in calcium regulatory proteins constitutes a switch modulating signaling and regulating apoptosis in mammal heart cells [40], and the redox status of two specific Met controls actin polymerization during development and immune response [41,42].

Regarding photosynthetic organisms, the report by Sanchez et al. [43] in 1983 provided the first evidence for MSR activity in extracts from various higher plants, the activity being largely localized in the chloroplastic fraction. The first molecular characterization of a plant *MSR* gene was performed in *Brassica napus* in 1996 [44], and the evidence for the participation of *MSR* genes in defense against oxidative stress was unveiled in *Arabidopsis thaliana* knockout mutants [45]. In the following years, numerous studies provided information about plant *MSR* genes and enzyme biochemical properties. In two previous reviews [46,47], we thoroughly described the organization of MSR families in photosynthetic organisms, which are more complex than in other organisms. Indeed, they include 14, 9, 8, and 8 members in Arabidopsis, poplar, maize and *Chlamydomonas reinhardtii*, respectively, against 2 and 4 in yeast and human, respectively [47,48]. These reviews also described the structure and sequence of *MSR* genes, the various catalytic mechanisms involved in the regeneration of the enzyme activity, and reported the first available data concerning the physiological functions of these reductases in the green lineage. At that time, plant MSRs were presumed to play mainly a direct antioxidant function to preserve cell structures via protein repair and avoidance of oxidative damage. Since then, as shown in other organisms, another key and exciting role in signaling pathways has emerged for plant MSRs. In this review, we present the current knowledge regarding MSRs in photosynthetic organisms. We focus more particularly on their subcellular localization and tissue distribution, on the stimuli and signaling actors controlling their expression, activity and substrate levels, Finally, we review the physiological functions and potential targets reported for plant MSRs in relation with antioxidant defense and signal transduction. 

## 2. Subcellular Localization and Organ Distribution of Plant MSRs

### 2.1. Subcellular Localization

**Figure 2:**
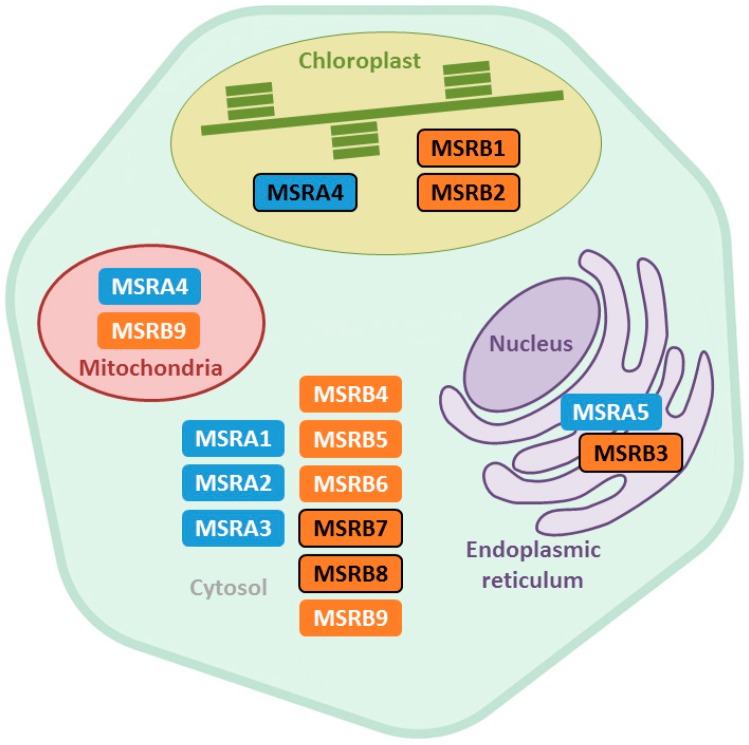
Subcellular localization of MSRs in *Arabidopsis thaliana*. Localization of MSRA4, MSRB1, and MSRB2 in plastid, of MSRB3 in endoplasmic reticulum and of MSRB7 and MSRB8 was proven experimentally [49,50,51,52]. These isoforms appear in black. For other isoforms (in white), the localization is based on predictions from sequence analysis or on proteomic analyses [63,64,65].

In Arabidopsis, the MSR family consists of 14 members, predicted to be localized in plastids, cytosol, and endoplasmic reticulum (Figure 2). The subcellular localization of some of them was proven experimentally, as reported for the three plastidial MSRA4, MSRB1, and MSRB2 isoforms [49,50], MSRB7 and MSRB8 in cytosol [51], and MSRB3 in endoplasmic reticulum [52]. Consistently, several MSRA and MSRB isoforms from other species (rice, tobacco, tomato, pepper, soybean, papaya, and banana) were found to localize in cytosol and plastids in Arabidopsis protoplasts using a bimolecular fluorescence complementation (BiFC) approach [53,54,55,56,57,58,59,60,61,62]. In other respects, data gained from BiFC experiments suggest that some cytosolic MSRA and MSRB isoforms from litchi and banana are present both in cytosol and in nucleus [61,62]. Taken collectively, these data indicate that MSRs are present in most subcellular compartments. Intriguingly, no MSR isoform is predicted to be addressed in mitochondria. Proteomic analyses performed on mitochondrial fractions found Arabidopsis MSRA4 and MSRB9 as potentially present in this compartment [63,64,65], but the double addressing of these isoforms remains to be experimentally validated. These data raise the question of how the Met redox status is preserved in plant mitochondria. Indeed, the maintenance of protein redox homeostasis is crucial in this organelle where ROS are produced as by-products in case of impairment of the electron transfer chain [66].

### 2.2. Organ Distribution

As inferred mainly from transcriptomic approaches in the plant model *A. thaliana* [46,47], *MSR* genes exhibit differential expression as a function of organ type. Briefly, *MSR4*, *MSRB1*, *MSRB2*, and *MSRB6* are specific of aerial photosynthetic organs while *MSRA2*, *MSRB5*, *MSRB7*, *MSRB8*, and *MSRB9* are preferentially expressed in root. Accordingly, *MSRB7* and *MSRB8* expression in root was confirmed by quantitative reverse transcription (qRT-PCR) analysis [51], and the high abundance of plastidial MSR proteins (A4, B1 and B2) in leaves was shown by Western analysis [50]. However, the three isoforms are also present in floral organs, and a relatively important amount of MSRA4 protein is detected in root [50], revealing the importance of investigating protein levels to gain an accurate overview about MSR distribution in plant organs and tissues. Regarding other species, data are still scarce at the protein level. Indeed, except the reports by In et al. [67] in rye leaves and by Châtelain et al. [68] in *Medicago truncatula* seeds, most studies are based on Northern, qRT-PCR or β-glucuronidase (GUS) expression analyses. Similarly to what reported in Arabidopsis, genes coding for plastidial isoforms are preferentially expressed in green organs. This is the case of pepper *MSRB2*, its transcript level being much higher in leaf, flower, and stem than in root [56]. In young *Glycine soja* seedlings, the messenger coding for plastidial MSRB5 is present in vascular tissues and two others coding for plastidial isoforms (B1 and B2a) are preferentially found in leaf and stem [60]. Another study in soybean indicated that four of the five *MSRB* genes are more expressed in leaf than in root or seed [69]. Similar differential patterns were reported in monocotyledons. For example, in rice, the *MSRB5* transcript level is higher in leaf than in other organs [70], and *MSRB1.1*, which codes for a plastidial isoform, is much more expressed in leaf and flower than in root or stem [55]. In contrast, the *MSRA4.1* transcript is detected at a similar level in all organs, although the encoded protein is localized in plastid [55]. Finally, an extensive qRT-PCR analysis in maize indicated that *MSRA2* and *A4* are mainly expressed in leaf and *MSRA5.1* and *A5.2* in seed [48]. Regarding *MSRB* genes, *MSRB1* and *MSRB2* are specifically expressed in leaf and *MSRB5.1* and *B5.2* in root [48]. In other respects, one strawberry *MSRA* gene is expressed only in the receptacle of red mature fruit [71]. Consistently with this finding, the expression of one *MSRA* gene is strongly up-regulated in the last stages of ripening and senescence in banana fruit [62]. In litchi fruit, the expression of *MSRA1*, *A2*, and *B1* genes decreases as senescence proceeds during storage [61].

These data indicate that MSRs are present in all plant organs, but display distinct expression patterns in many cases. They need to be deepened to determine whether transcript and protein abundances are correlated and to delineate expression in specific tissues and organs such as floral components, for which the knowledge is still scarce. By crossing the data related to subcellular and organ localization, it turns out that several cytosolic MSRs are specifically expressed in root while plastidial MSRs are preferentially found in aerial photosynthetic organs where photosynthesis takes place. This could mean that the function of the latter is linked to this metabolism, the activity of which can lead to the production of ROS altering redox homeostasis. On the other hand, and as mentioned earlier, MSRs fulfill key roles during ageing in bacterial, yeast, and animal cells. The expression patterns of plant genes reveal that plastidial MSRBs are more abundant in young leaves than in older ones [50], and that other isoforms are specifically expressed in fruit and seed at different stages of the ripening or maturation processes [61,62,68,71]. These findings are consistent with the participation of MSRs in the control of ageing and senescence processes in plant organs.

## 3. Regulation of the Expression of *MSR* Genes in Photosynthetic Organisms

### 3.1. Effect of Environmental Conditions

In agreement with the biochemical function of MSRs in the maintenance of Met redox status, the first microarray data gained in Arabidopsis revealed that environmental constraints leading to oxidative stress result in increased expression of most *MSR* genes [46]. In the last years, the expression patterns of these genes have been refined in various types of photosynthetic organisms in relation with abiotic, but also biotic constraints. The data gained in higher plants are summarized in Table 1.

Consistently with the data reported in Arabidopsis, oxidative stress conditions generated by manganese deficiency or copper excess in *C. reinhardtii* and *U. fasciata* algae lead to up-regulation of *MSRA* genes [72,73]. In *C. reinhardtii*, the expression of three *MSRB* genes (*1.1*, *1.2* and *2.1*) is induced in very high light conditions and upon treatment with H_2_O_2_ [74]. Data that are more meaningful regarding the physiological factors regulating expression of *MSR* genes were gained in relation with the activity of the photosynthetic chain. In *U. fasciata*, the transcript levels of *MSRA* and *MSRB* genes peak following 1-h light exposure [75]. Most interestingly, the use of various inhibitors of the photosynthetic electron chain revealed that the expression of these genes differentially depends on the redox status of components belonging to the cytochrome *b_6_f* complex or downstream complexes. This strongly supports the hypothesis that the photosynthetic activity level, which modulates plastidial redox homeostasis, plays an essential role in pathways regulating the expression of *MSR* genes. As these genes are nuclear-encoded, these pathways very likely involve retrograde signaling from plastid to nucleus. 

Numerous studies report that oxidative stress conditions are associated with up-regulation of *MSR* gene expression in higher plants (Table 1). Thus, MV treatment leads to increased transcript or protein levels of tobacco MSRB3, tomato MSRA2, A4 and A5, rice MSRB1, rye MSRA, and Arabidopsis MSRA4, MSRB7, and MSRB8 [51,55,58,67,76,77]. More physiological constraints that impair the cell redox homeostasis enhance *MSR* expression. For instance, copper excess leads to *MSRB5* up-regulation in rice [70]. In Arabidopsis, exposure to cadmium triggers the antioxidant defense system, notably the expression of most *MSR* genes, but also provokes a decrease in the abundance of plastidial MSRBs [78,79]. In *Brassica juncea*, such a treatment results in a higher amount of cytosolic MSRA2 [80]. In other respects, increased amounts of plastidial MSRs (A4, B1 and B2) were observed in Arabidopsis plants exposed to photooxidative stress conditions generated by high light and low temperature conditions [50]. In rye, high light conditions induce the accumulation of a cytosolic MSRA protein [67].

Regarding other environmental constraints, many studies reported increased *MSR* expression in conditions leading to osmotic stress such as water shortage, high salt, and low temperature (Table 1). This was first established from microarray data in the Arabidopsis plant model [46,47]. In maize, most *MSR* genes are up-regulated in root, stem and leaf in the presence of polyethylene glycol (PEG) or NaCl with distinct kinetics depending on gene type and organ [48]. Consistently, in rice, *MSRA4.1* and *MSRB1.1* expression is enhanced by mannitol, high salt and low temperature [55]. In other respects, a higher MSRA protein abundance was observed in cold-hardened rye plants [67] and in maize seedlings, low temperature induces the expression of *MSRA5* in mesocotyl [81]. In soybean, Chu et al. [69] reported differential expression of the five *MSRB* genes in response to drought and high salt. Most importantly, they observed that three of them (*MSRB2*, *B3* and *B5*) show increased transcript levels in response to drought, but only in leaf and at distinct vegetative or reproductive stages. In tobacco, *MSRA4* expression is up-regulated by dehydration and cold, but not modified by high salt [59], whereas that of *MSRB3* is enhanced by cold and salt [58]. Finally, in tomato, *MSRA3* and *MSRA4* are substantially up-regulated by mannitol, high salt, and low temperature [82]. Altogether, these data give strong credence for essential functions of MSRs in plant responses to osmotic constraints. Accordingly, in the *Atriplex halimus* halophyte species, cultivation in the presence of 300 mM NaCl increased the abundance of plastidial MSRA concomitantly to a higher total MSR activity in a salt-tolerant genotype compared to a salt-sensitive one [83]. However, in barley, an increased protein amount of one MSR isoform was noticed using a proteomic approach in a salt-susceptible genotype compared to a salt-tolerant one [84], and no difference was noticed in the amount of plastidial MSRs in two cultivars exhibiting contrasted response to water deficit [85]. In other respects, exposure to carbonate, which induces alkaline stress in addition to osmotic and ionic stresses, leads to the expression of most *MSRB* genes in *Glycine soja* whether in leaf or in root [60].

In comparison, less is known regarding the expression of *MSR* genes in response to biotic stress. The first evidence was provided in Arabidopsis plants that display a strongly increased *MSRA4* transcript level following infection by the cauliflower mosaic virus, but no change in response to a virulent *Pseudomonas syringae* strain [49]. In papaya, infection by the ringspot virus leads to up-regulation of *MSRB1* expression in the late stages [54]. Most interestingly, some *MSR* genes could be involved in plant immune responses. Thus, in poplar leaves, the abundance of a plastidial MSRB is unchanged during infection by an incompatible rust *M. larici-populina* strain, whereas the protein level increases in the presence of a compatible strain. In contrast, the amount of another MSRB strongly decreases after infection either with compatible or incompatible fungi [50]. In pepper, the level of a transcript coding for a plastidial MSRB isoform first strongly decreases following infection both with compatible and incompatible *Xanthomonas axonopodis* strains, and then is restored to the initial level only in the case of the compatible reaction [56]. In *A. thaliana*, avirulent and virulent *P. syringae* strains lead to very distinct expression patterns for *MSRB7* and *MSRB8*, both being much more strongly up-regulated in the case of an incompatible reaction [86]. Moreover, an increased *MSRA2* transcript level was noticed early following infection of Arabidopsis seedlings by the parasite plant *Orobanche ramosa* [87]. Altogether, these data indicate that MSRs likely participate in immunity mechanisms and active defense against most types of biotic constraints, to which plants are exposed.

### 3.2. Signaling Actors Involved in the Control of MSR Gene Expression

#### 3.2.1. Involvement of ROS and Reactive Nitrogen Species (RNS) in MSR Gene Expression

Most, if not all environmental conditions, leading to the expression of *MSR* genes reported in the previous section, involve the production of ROS due to metabolic impairment and subsequent changes in cell redox homeostasis that are associated with specific ROS signatures [88]. Reactive nitrogen species (RNS) constitute another type of oxidant molecules, tightly related to ROS, such as peroxynitrite produced by the reaction of nitric oxide (NO) with superoxide. Both ROS and RNS are deleterious at high level since they damage all macromolecules through oxidation, but fulfill critical signaling functions at basal level in developmental processes and responses to environmental constraints [89,90]. ROS and RNS are produced in very specific subcellular cell compartments, and very likely do not directly regulate gene expression at the transcriptional level due to their site of production within cell, diffusion properties and half-life time [2]. They are assumed to initiate or transfer signaling information through redox metabolic reactions with antioxidant molecules, lipids, and proteins [90,91]. Based on the increasing knowledge gained in bacterial, yeast, animal, and plant models, ROS/RNS are now considered as signals shifting cell redox homeostasis and major drivers in responses and adaptation to abiotic and biotic constraints [91,92]. For instance, the role of ROS is critical in the control of cell death upon incompatible reaction in plants [93].

Thereby, ROS and RNS species are good candidates to control *MSR* expression in photosynthetic organisms. Consistent with this hypothesis, Chang et al. [94] observed in Chlamydomonas distinct expression patterns for five *MSR* genes in high light conditions, four being up-regulated (*MSRA3*, *A5*, *B2.1* and *B2.2*) and the fifth down-regulated (*MSRA4*). Using various ROS scavengers and generators, they showed that these patterns are specifically linked to the ROS type. For example, hydrogen peroxide and superoxide differentially modulate *MSRB2.1* and *MSRB2.2* expression, respectively. Regarding RNS, a similar pharmaceutical approach in *U. fasciata* showed that NO induces acclimation to high light concomitantly to upregulation of *MSRA* and *MSRB* expression [95].

In higher plants, exposure to MV leads to up-regulation of various *MSR* genes in many species [51,55,59,67,76,82]. Treatment with H_2_O_2_ leads to more complex data since a decreased *MSRA4* transcript amount is noticed in tobacco seedlings [59] while the four tomato *MSRA* genes display contrasted responses [82]. In Arabidopsis, two *MSRA* genes, out of the four tested, display enhanced expression following H_2_O_2_ treatment [96]. Accumulation of singlet oxygen, a ROS produced when chlorophyll triplets excite O_2_, induces *MSRB7* expression in Arabidopsis [97]. In other respects, priming with NO prevents up-regulation of *MSR* expression in cadmium-treated Arabidopsis plants, very likely due to reduction in ROS production and limitation of oxidative stress [78]. Altogether, these data reveal that ROS-related signals control the expression of *MSR* genes in higher plants. Although experimental evidence remains scarce, RNS also very likely regulate *MSR* expression as illustrated by the strong *MSRB7* up-regulation in Arabidopsis plants treated with *S*-nitrosoglutathione, a reservoir for NO [98].

#### 3.2.2. Involvement of Phytohormones in MSR Gene Expression

Tight and complex interplays between ROS-dependent transduction pathways and other recognized signaling components like phytohormones, mitogen-activated protein kinases and calcium ions are established [99]. With regard to *MSR* genes, this is illustrated by the analysis of linolenic acid-responsive genes in Arabidopsis cell cultures subjected to osmotic stress [100]. Using a RNA-seq approach, this study reported that *MSRB7* and other antioxidant genes are strongly induced by linolenic acid, which is released from plastidial membrane galactolipids and is a precursor of jasmonic acid (JA). JA, together with derivatives such as methyljasmonate (MeJA), constitute a phytohormone family derived from oxidized lipids (oxylipins) and mediating responses to conditions modifying the redox homeostasis, notably biotic constraints [101]. In tomato seedlings treated with JA, the expression of the four *MSRA* genes is either down-regulated or unchanged [82]. However, in Arabidopsis, JA treatment increases the transcript level of two *MSRA* genes and decreases this level for two others [96]. In pepper, a strong decrease in *MSRB2* transcript level was observed following exposure to MeJA and to salicylic acid (SA) [56]. SA, another key hormone involved in plant immune responses to microbial pathogens [102], strongly triggers the expression of one *MSRA* gene in tomato, but decreases the expression of another [82].

Among phytohormones, abscisic acid (ABA) is central to plant responses to osmotic constraints such as drought or high salt partly through the transcriptional control of gene expression [103]. In many plant species, up-regulation of *MSR* expression was reported upon these constraints (cf. Section 3.1). Accordingly, exposure of plants to ABA triggers *MSRA4* expression in Arabidopsis [96], *MSRB2* in soybean [69], and *MSRB3* in tobacco [58]. Nevertheless, other *MSR* genes from these species and tomato are unresponsive to ABA or their expression is down-regulated by this phytohormone [59,69,82,96], revealing specific ABA-dependent patterns.

Phytohormones are well known actors in the control of plant development. Most importantly, ROS and thiol-based mechanisms are also essential signaling players allowing proper development of plants in relation with varying environment and energy availability [10]. As mentioned in Section 2.2, some *MSR* genes are specifically or highly expressed in fruits, such as banana and strawberry [61,62,71]. Climacteric fruits undergo ripening following harvest, a process during which the phytohormone ethylene plays a critical role. Interestingly, in the climacteric banana fruit, *MSRA7* expression is up-regulated during ripening and dramatically increased following ethylene treatment [62]. Consistently, the *MSRA2* transcript level is strongly up-regulated in another climacteric fruit, tomato, following treatment with ethephon, an ethylene-releasing compound [82]. In other respects, the transcript amounts of three *MSR* genes decrease in litchi, a non-climacteric species, along with the senescence process following harvest [61]. These findings suggest that the expression of *MSR* genes depends on fruit developmental stage and is controlled via the action of phytohormones, such as ethylene, which regulate maturation and senescence processes.

#### 3.2.3. Conclusions

Based on all available data, we conclude that in higher plants ROS play a central role in the control of the expression of *MSR* genes at the transcriptional level upon environmental constraints. This is consistent with the role of MSR enzymes in the maintenance of protein redox status and the up-regulation of the expression of *MSR* genes generally observed upon these constraints (Table 1). ROS transfer signaling information through redox metabolic reactions with different compounds, and participate in transduction pathways involving other actors such as phytohormones [10,90]. Interestingly, phytohormones play more complex roles in regulating *MSR* gene expression, as mentioned above and shown in Table 1, probably in relation with both environmental condition and development stage.

## 4. MSR Activity and MetO Content in Higher Plants

### 4.1. MSR Activity in Plant Extracts

Another question emerges from the subcellular distribution of MSRs: do they display similar abundance and activity? The pioneer works by Sanchez et al., and Ferguson and Burke [43,104] indicated that in various plant species a large part of the leaf MSR activity (85%) is localized in the chloroplastic fraction, the remaining 15% being measured in the cytosol. Surprisingly, the number of MSR isoforms is low in this compartment: 3 out of 14 in Arabidopsis. Using a genetic approach i.e., mutants knockout for *MSRB1*/*MSRB2* genes and/or knockdown for *MSRA4* expression, we confirmed this finding by showing that the two plastidial MSRBs account for the greater part of leaf MSR capacity (75%), and that this capacity is further decreased in plants deficient in the three plastidial isoforms [105,106]. These data reveal that MSRAs and MSRBs do not fulfil equivalent roles in terms of activity and physiological function, at least in chloroplast, and suggest that the two MetO diastereoisomers are not generated in a racemic proportion *in planta* [106]. The predominant plastidial MSR capacity in leaf is very likely related to the fact that chloroplast is a major site of ROS production because of the photosynthetic electron transfer chain activity in light conditions [66]. Interestingly, the MSR activity is in the same range from 10 to 80 pmol Met·min^−1^·mg·prot^−1^ in Arabidopsis root, stem, leaf, flower bud, flower, green silique, and seed extracts ([68,105]; our unpublished data). The contribution of each MSR isoform likely depends on organ type and physiological context. Accordingly, the MSR capacity in Arabidopsis lines lacking plastidial MSRBs is much less decreased in seed than in leaf [68,105] and the MSR activity in an Arabidopsis mutant lacking cytosolic *MSRA2* is specifically and strongly decreased by 50% in the second part of the dark period compared to wild-type (WT) [107]. Thorough measurements of MSR activity at the organ level in mutants knockout for the various *MSR* genes would greatly help to delineate the physiological functions of all isoforms.

Relatively few data are available concerning the effects of external factors on MSR activity in plants. Ferguson and Burke [108] measured the activity in various species subjected to high temperature or water deficit and observed species-dependent changes. For instance, water shortage leads to decreased and increased activity in cotton and pea, and in wheat, respectively. In Arabidopsis, photooxidative constraints result in increased MSR activity in chloroplastic or leaf fractions [76,105], and treatments with NaCl or ABA, but not exposure to low temperature, provoke substantial reductions in activity [96,105]. In wild soybean, moderately and strongly increased activities were noted upon high salt (NaCl) or carbonate alkaline stresses, respectively [60]. In *A. halimus*, high salinity provokes a noticeable increase in MSR activity in a tolerant genotype, but not in a sensitive one [85]. However, in barley, no difference in activity was recorded in two contrasted cultivars for water shortage tolerance [86]. In conditions of biotic stress, no significant change in MSR activity was observed in tomato plants challenged with Phytophtora [56].

### 4.2. MetO Content in Plants

Regarding protein-bound MetO, the substrate of MSR, its proportion in relation to the total quantity of Met and MetO is in the range from 5 to 20% in various species (pea, wheat, potato, Arabidopsis) grown in optimal conditions [45,76,108]. However, much lower levels (less than 1.5%) were also measured in Arabidopsis [52,105]. Interestingly, the MetO quantity varies during the day/night cycle, with a 4-fold higher content in the middle of the light period [109]. Genetic studies confirmed the biochemical function of MSRs via the determination of MetO content in extracts from modified plants. Arabidopsis mutants deficient in various types of MSRBs or MSRAs exhibit a higher MetO level compared to WT plants [45,52,76,105,109]. The increase in MetO proportion was recorded mainly in plants subjected to environmental constraints such as high light or low temperature [52,105]. Consistently, Arabidopsis plants overexpressing plastidial MSRBs or cytosolic MSRB3 display a lower protein-bound MetO level upon photooxidative constraints [52,76,105].

The consequences of environmental variations on MetO content in non-modified plants are less simple to interpret. In various species subjected to high temperature or water deficit, no great variation was noticed, except in pea plants exposed to high temperature where a strong decrease was noticed [108]. In 6-week old Arabidopsis WT plants, we reported a decreased peptide-bound MetO content upon high light and long photoperiod conditions, but no variation at low temperature [105]. However, other studies reported substantial increases in the peptide-bound MetO proportion in Arabidopsis plants subjected to severe photooxidative stress [45,76], or during cold acclimation [52].

### 4.3. Signals Involved in the Control of MSR Activity and MetO Level

Taken collectively, these findings reveal that in physiological conditions MSR activity and MetO content are regulated in a fine and complex manner in plant cells as a function of species type, environmental condition and stress intensity. Higher MSR activity and MetO level are generally recorded upon constraints leading to pronounced oxidative stress, consistent with the fact that Met oxidation occurs in the presence of ROS excess, and is a marker of protein damage [2]. Accordingly, a proteomic study of Arabidopsis *catalase 2* knockout plants exposed to very high light, identified more than 50 proteins displaying a higher MetO content compared to WT, due to deficiency in H_2_O_2_ scavenging [110]. However, another proteomic study on Arabidopsis cell cultures revealed that Met oxidation in proteins could result from the action of non-oxidative signaling molecules [111]. Thereby, it can be concluded that MetO formation in proteins even occurs in the absence of ROS excess during environmental changes and is a very finely controlled PTM mediated by interplaying transduction pathways and actors remaining to be unveiled.

## 5. Physiological Functions of Plant MSRs

### 5.1. Oxidative Treatments and Photooxidative Conditions

In animal, yeast and bacterial cells, many studies revealed the involvement of MSRs in the responses to MV or H_2_O_2_ [33,36,38]. In a first step, a similar physiological function has been searched in photosynthetic organisms. The use of knockdown and overexpression *C. reinhardtii* lines showed that two MSRB isoforms play specific protective roles against oxidative stress generated by H_2_O_2_ or high light [74]. Following exposure to MV, ozone or high light intensity, *MSRA4*-antisense or -overexpression Arabidopsis lines display poorly or better-preserved photosynthetic activity, respectively, compared to WT [76]. Moreover, these phenotypes are associated with increased or decreased MetO content, indicating that MSRA4 likely plays a role in the protection of photosynthetic structures against oxidative damage. Consistently, based on chlorophyll content, ion leakage and growth measurements, improved tolerance towards MV and H_2_O_2_ treatments was noticed in Arabidopsis seedlings overexpressing cytosolic MSRB7 or MSRB8 isoforms grown either on synthetic media or on soil, while a greater sensitivity to these compounds was observed in deficient lines [51]. Tomato plants ectopically expressing Arabidopsis MSRB7 also display increased tolerance to MV [51]. Interestingly, *MSRB7*- and *MSRB8*-overexpressing Arabidopsis plants exposed to MV exhibit reduced H_2_O_2_ level and increased glutathione-*S*-transferase (GST) activity, clearly showing the integration of MSRs in the antioxidant network. Based on the higher susceptibility to MV of Arabidopsis plants lacking MSRB3, this isoform located in endoplasmic reticulum is also presumed to preserve cell structures against oxidative damage [52]. In rice, decreased tolerance to MV and copper excess was reported in plants lacking the MSRB5 isoform, with both treatments leading to severe oxidative stress [70].

### 5.2. Abiotic Constraints

In natural environments, most abiotic constraints induce changes in cell redox homeostasis to a lesser extent than those described above following exposure to strong oxidizing agents, which are not relevant from a physiological point of view. The functions of MSRs in more meaningful conditions are thus somewhat harder to determine, and their expression patterns are very helpful to progress in this direction. Therefore, based on *MSRB3* induction in *A. thaliana* at low temperature, Kwon et al. [52] showed that a mutant deficient in this isoform loses the ability to tolerate freezing temperatures following cold acclimation. Overexpression of a mutated active version of the *MSRA4* gene, which is induced by NaCl treatment, results in enhanced tolerance to high salt in in vitro grown seedlings [112]. Regarding tolerance to heavy metals, ectopic expression of *Brassica rapa MSRA3* in Arabidopsis leads to better growth of in vitro plantlets in the presence of 50 µM cadmium [113]. Other, less severe abiotic constraints, but applied for a long time, have been reported to impair the growth of Arabidopsis lines lacking MSR isoforms. Thus, plants deficient in both plastidial MSRB1 and MSRB2, display a rosette weight reduced by ca. 25% compared to WT when grown at 10 °C for 18 days [105]. These plants also exhibit substantially reduced growth when continuously cultivated in long day/high light conditions compared to short day/moderate light [105]. In other respects, the growth of an Arabidopsis line lacking cytosolic MSRA2 is impaired in short-day and not in long-day conditions [107]. MSRA2 may limit the oxidative damage occurring in proteins at the end of a long dark period [105]. The long duration—several weeks in these experiments [105,107]—allowed to uncover physiologically relevant functions for plant MSRs, probably due to the fine and timely control of MetO formation in proteins as a function of environment and developmental stage. 

In species other than Arabidopsis, several MSRs fulfil essential roles in responses to osmotic constraints. Thus, in rice, overexpression of plastidial MSRA4.1 is associated with improved tolerance to high salt (300 mM NaCl), as inferred from photosynthetic activity and oxidative damage measurements [55]. Furthermore, rice plants overexpressing pepper *MSRB2* exhibit better tolerance to water deficit than WT upon shortage and higher survival rate following re-watering [57,114]. In these plants, microarray analysis indicated that genes coding for photosystem components are much less down-regulated in drought conditions than in WT [57]. Regarding salt stress, few data are available regarding the protective effect of MSRs. Recently in *G. soja*, their involvement was reported in a specific constraint, i.e., high carbonate that leads to alkaline stress. Indeed plants overexpressing *MSRB5a.1* exhibit better tolerance to high carbonate either at the germination stage in vitro or during vegetative growth on soil [60].

### 5.3. Biotic Constraints

With regard to biotic constraints, two reports clearly provided physiological evidence for a role of plant MSRs upon attack of fungi or bacteria. Overexpression in tomato of pepper *MSRB2* results in enhanced resistance to two Phytophtora species that cause severe diseases in Solanaceae [56]. In parallel, these tomato lines display reduced H_2_O_2_ content following infection and are more tolerant to oxidative stress generated by high light or MV. This study also showed that pepper lines silenced for *MSRB2* expression exhibit increased ROS production, accelerated cell-death in the case of incompatible infection by a bacterial Xanthomonas race and increased susceptibility following infection by a virulent strain [56]. Data confirming the essential roles of MSRs in immune responses were also recently reported in Arabidopsis [86]. Compared to WT, knockout or overexpression lines for cytosolic *MSRB8* display increased sensitivity or tolerance, respectively, to an avirulent strain of *Pseudomonas syringae* while no modification in the responses of transgenic plants occur following infection by virulent strains [86]. Altogether, these data demonstrate that plant MSRs fulfill critical functions in plant immune mechanisms that could be explored for improving crop resistance.

It is worth mentioning that the plant pathogen *Erwinia chrysanthemi* when deficient for *msrA* displays reduced pathogenicity, revealing the requirement of this gene for the full virulence of the bacteria, likely via the participation in defense mechanisms against plant-produced ROS [115]. This finding and those reported on plant MSRs in compatible and incompatible reactions show that MSRs are essential actors in defense mechanisms in both partners during the infection process. Further, plant MSRs could even constitute targets of pathogens. Indeed, in papaya, the Nla-pro protein of the papaya ringspot virus interacts with the preprocessed MSRB1 protein and prevents its import in chloroplasts, thus possibly weakening antioxidant defenses of the host plant [54].

### 5.4. Involvement in Ageing Process

The participation of MSRs in ageing and lifespan control is well established in bacteria, yeasts, and animals [33,37]. The only evidence in plants concerns seed viability. Seeds are in a natural oxidative context leading to protein oxidation that at high level is deleterious and associated with ageing. In two *Medicago truncatula* genotypes contrasted for seed quality, a strong positive correlation was observed following controlled deterioration between the time to a 50% drop in viability and the MSR capacity of mature seeds. A similar correlation was recorded in seeds of *A. thaliana* lines, altered for *MSR* gene expression and capacity [68]. These data clearly reveal that the MSR repair system plays a critical role in the establishment and preservation of longevity in plant seeds.

## 6. Mode of Action and Substrates of Plant MSRs

**Figure 3:**
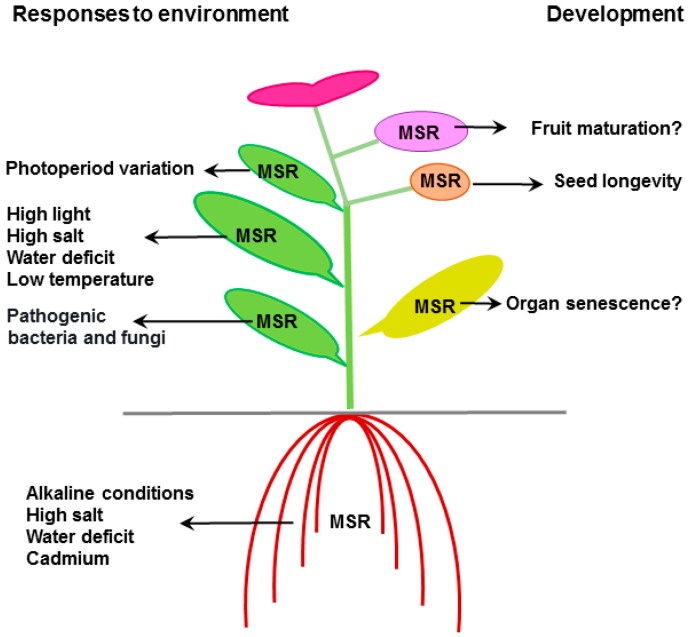
Physiological functions of plant MSRs. The functions of MSRs in the responses to high light, high salt, high carbonate, water deficit, low temperature, cadmium and in photoperiod adaptation was shown in Arabidopsis [50,52,76,105,107,112,113] rice, [55,57,114] or *Glycine soja* [60]. The participation in responses to biotic constraints was reported in Arabidopsis [86], pepper, or tomato [56] the involvement in seed ageing in Arabidopsis and *Medicago truncatula* [68]. The presumed role in organ senescence and fruit maturation is based on expression patterns and identification of possible partners in Aarabidopsis, strawberry, litchi, and banana [50,61,62,71].

Upon environmental constraints and development (Figure 3), plant MSRs likely fulfil an antioxidant function through MetO repair in proteins exhibiting accessible Met residues positioned on their surface [22,38]. Besides, the increasing knowledge gained recently gives strong credence for much more specific functions in the control of Met redox status in particular proteins involved in signaling pathways. Therefore, the identification of MSR partners is a prerequisite for giving accurate insight into the physiological roles of these reductases in plants. In recent years, in addition to targeted approaches, several global strategies based on biochemical, transcriptomic, and proteomic methods were set up to search for proteins interacting with MSRs, or displaying MetO residues, as well as genes exhibiting modified expression in plants up- or down-regulated for *MSR* genes.

### 6.1. Strategies for Searching Plant MSR Targets

#### 6.1.1. Proteins Displaying High Met Content

One of the first strategies to identify MSR targets in plants is based on the hypothesis that proteins exhibiting a high Met percentage or Met-rich domains are good candidates. The Arabidopsis plastidial small heat shock protein of 21 kDa (HSP21) possesses a unique 19-residue domain carrying six Met in an amphipathic helix. In vitro, MSRA4 counteracts Met oxidation in HSP21, restores its oligomeric conformation and maintains its chaperone-like activity [116]. Similarly, based on the high Met content (4.6%) and on the fact that its homologue in *Escherichia coli* is a target of MSRs [117]. cpSRP54, the chloroplast signal recognition particle of 54 kDa that addresses light-harvesting complex (Lhc) proteins to thylakoids, was presumed to be a target of MSRs. Using recombinant forms, we showed that oxidized cpSRP54 is a substrate for plastidial MSRBs [105]. Interestingly, the oxidized form of the other component of the signal recognition particle, cpSRP43 that exhibits a lower Met content (1.9%) is also reduced by MSRBs [105]. More recently, a thorough survey of genomic data allowed identifying Met–rich proteins, MRPs, far more systematically in Arabidopsis and soybean. The search based on two criteria, peptide length of at least 95 residues and Met content higher than 6%, resulted in the isolation of 121 and 213 genes, respectively, coding for proteins meeting both conditions [118]. Of note, the function of 50% of encoded proteins is unknown. Such *in silico* approaches sound very promising and powerful to search proteins harboring Met-rich domains and identify physiological targets of MSRs.

#### 6.1.2. Proteins Exhibiting Modified MetO Content in Response to Oxidative Treatments or Signaling Molecules

The proteins carrying MetO residues are also likely to be MSR substrates. Unfortunately, there is no efficient tool for rapidly isolating such proteins, notably due to the lack of highly reliable antibodies specific to MetO [119] to set up immunological-based analyses at the proteome scale. In the plant field, there is only one example reporting the use of these antibodies to search for MetO-containing proteins. The comparison of Western patterns from rice plants either WT or overexpressing pepper plastidial MSRB2 highlighted a higher signal level in the range of 40 kDa in WT. Mass spectrometry analysis identified 3 proteins located in plastid: porphobilinogen deaminase (PBGD), dihydrodipicolinate reductase I and ferredoxin-NADP reductase. Based on the MetO content of recombinant PBGD following H_2_O_2_ treatment and its ability to be reduced by pepper MSRB2, PBGD was proposed as a physiological substrate of MSRs [57]. To overcome the low specificity of MetO antibodies, other strategies based on redox proteomics were set up. Marondedze et al. [111] used titanium oxide treatment in combination with dihydroxybenzoic acid to enrich MetO containing peptides. They analyzed protein extracts of Arabidopsis cell suspension cultures treated with an analogue of cyclic guanosine monophosphate (cGMP), and identified by tandem mass spectrometry 94 and 224 unique proteins carrying MetO strongly enriched following 30 and 60 min of treatment, respectively. However, it is important to note that such a method of enrichment may provoke non-physiological oxidation of Met [120]. Another approach based on tandem-mass spectrometry was developed to isolate proteins differentially oxidized in WT and *MSRB7*-overexpressing Arabidopsis plants treated with MV [121]. It consisted to treat the protein extract with cyanogen bromide that hydrolyses the C-ter of Met, but not of MetO, before trypsin digestion and protein identification. This analysis identified more than 30 proteins that could be MSR substrates *in planta* [121]. Another strategy to identify proteins carrying MetO consists to take advantage of recombinant MSRs [110]. This combined fractional diagonal chromatography (COFRADIC) is made of three steps: (i) HPLC-fractionation of peptides; (ii) treatment of peptides with recombinant MSRs, which induces a hydrophobic shift in MetO containing peptide; and (iii) refractionation and identification of shifted peptides. Using this strategy, about 400 proteins carrying MetO were identified and differential oxidation was investigated in extracts from WT and from *catalase 2* knockout plants that over-produce H_2_O_2_ at ambient CO_2_ concentration. Consistently, 51 proteins were significantly more oxidized at the level of Met in this genetic background [110]. These three studies clearly show the power of redox proteomic to identify potential physiological targets of plant MSRs at a large scale and indicate that these reductases very likely possess a broad range of substrates.

#### 6.1.3. Proteins Interacting with MSRs

Proteins that interact with MSRs are also appropriate candidates to be reduced by these enzymes. Based on this hypothesis, we aimed at isolating plant MSR partners using affinity chromatography [122]. Using Arabidopsis recombinant MSRB1 and leaf extracts, we isolated 24 proteins, 13 being plastidial and potential physiological substrates of MSRB1. The other 11 could interact with non-plastidial MSRB isoforms. Several are actually substrates of MSRs when their recombinant forms are treated with H_2_O_2_ and others possess homologues in yeast or mammals known to interact with MSRs and/or to possess Met sensible to oxidation, arguing for the relevance of the affinity-based strategy to target physiological partners of MSRs in plants.

#### 6.1.4. Genes Displaying Modified Expression in Lines Up- or Down-Regulated for *MSR* Expression 

Proteins exhibiting differential abundance in plants down- or up-regulated for *MSR* expression are speculated to be MSR partners due for instance to decreased stability resulting from change in MetO content. Nonetheless, such proteins could participate in signaling pathways or metabolic processes altered by changes in the Met redox status. Comparative analysis of extracts from salt-treated WT or *MSRA4*-overexpressing Arabidopsis seedlings by two-dimensional electrophoresis coupled to mass spectrometry analysis identified five proteins with lower intensity in the modified line, among which two HSP70 isoforms [112]. A similar approach, pointed 9 proteins, including six located in plastid, that are more abundant upon cold-treatment in WT than in a *MSRB3*-deficient mutant [52]. Since MSRB3 is localized in reticulum endoplasmic, this isoform might participate in a transduction pathway regulating plastidial metabolism. In other respects, micro-array analysis indicated that overexpression of the pepper plastidial *MSRB2* in rice plants preserves the expression of numerous genes coding for photosynthetic proteins upon drought stress compared to WT [57].

Taken collectively, the data gained from different and complementary approaches for isolating MSR partners and targets in plants have led to the identification of numerous potential substrates. Nevertheless, most need to be validated from a biochemical point of view with regard to their capacity to be reduced by MSRs using reconstituted systems and recombinant proteins [105,122]. Further, investigations based on two-hybrid system in yeast and BiFC assays in plant cells will help to confirm their ability to interact in vivo with MSRs [61,62]. These approaches will have to be completed by redox proteome-scale analyses using plants modified for the expression of each *MSR* gene in relation with organ development and environmental conditions.

### 6.2. Identity and Functions of MSR Partners or Possible Targets 

From the data acquired using the various strategies described in the previous sections, we can discuss how MSRs fulfill their functions in line with the identity of their proven or putative partners and the phenotype of modified plants.

#### 6.2.1. Translation and Folding of Proteins

The plastidial elongation EFtu factor interacts with MSRB1 [122], and is less abundant in cold conditions in an *msrb3* mutant than in WT [52]. Further, recombinant EFtu is actually a substrate of MSRBs following treatment with H_2_O_2_ [122]. Consistently with these findings, proteins involved in translation are significantly enriched among the proteins prone to Met oxidation in *catalase 2*-deficient Arabidopsis plants [110]. Heat shock proteins (HSPs) or chaperones fulfill critical roles in proper folding of proteins, particularly in stress conditions. HSPs were among the first plant proteins presumed to be MSR substrates due to the presence of Met-rich domains in some like HSP21 [116]. Interestingly in Arabidopsis, other chaperone types are putative MSR targets, such as the chaperonin 60β and one heat shock cognate (HSC70-3) isolated using affinity chromatography [122], and one HSP70, the abundance of which is decreased in *MSRA4*-overexpressing plants [112]. Further, HSP70, HSP70B, and HSC70-2 are less oxidized at the level of Met in *MSRB7*-overexpressing plants [121]. As MetO is more hydrophilic than Met, MSRs would maintain the hydrophobic character of HSP regions binding unfolded proteins, thus preventing their aggregation [123].

From these reports, we conclude that plant MSRs are essential components preserving the activity of actors participating in proper elongation and folding of proteins. However, another hypothesis could be put forward in the sense that MSRs might associate to the complexes ensuring proper protein biogenesis and process, since yeast MSRs preferentially reduce MetO in unfolded proteins, protecting them from oxidative unfolding [20].

#### 6.2.2. Chlorophyll Metabolism and Photosynthetic Activity

Numerous proteins involved in photosynthesis are potential MSR substrates. For instance, the two cpSRP43 and cpSRP54 components of the chloroplastic signal recognition particle, which targets Lhc proteins to thylakoids, are efficiently reduced by plastidial MSRBs [105]. The porphobilinogen deaminase catalyzes the polymerization of four monopyrrole units into a linear tetrapyrrole intermediate necessary for the formation of chlorophyll and heme. The recombinant form of this protein harbors two Met residues that are prone to oxidation upon H_2_O_2_ treatment, and is reduced by MSRs [57]. Non-targeted approaches confirmed the importance of MSRs regarding photosynthetic structures. Among the 24 proteins interacting with Arabidopsis plastidial MSRB1, six are involved in photosynthetic processes: ATPase subunits, RuBisCO, RubisCO activase, phosphoribulokinase, and glyceraldehyde-3-phosphate dehydrogenase B [122]. In other respects, in rice plants subjected to water deficit, a much more pronounced down-regulation of the expression of photosynthetic genes, such as those coding for photosystem I (PSI) subunits, was observed in WT than in *MSRB2*-overexpressing lines [57]. The authors concluded that MSRB2 maintains chloroplast function through the repair of Met upon water deficit and modulates retrograde signals involved in the regulation of gene expression in nucleus. Based on these data in rice [57] and those showing that an Arabidopsis mutant lacking plastidial MSRBs exhibits delayed growth and decreased photosynthesis in high light or low temperature conditions [105], these roles appear essential under environmental constraints impairing photosynthesis and plastidial redox homeostasis. From all these studies, we conclude that MSRs likely preserve photosynthetic structures along the whole process from pigment biogenesis and light capture to carbon assimilation.

In other respects, in cold conditions several photosynthetic proteins (RubisCO, sedoheptulose-1,7-bisphosphatase, SBPase, and photosystem II oxygen-evolving complex subunits) are less abundant in an *msrb3* mutant than in WT [52]. These proteins are not targets of MSRB3 that is located in endoplasmic reticulum. Similarly, RubisCO, SBPase, RubisCO activase, and carbonic anhydrase are less oxidized at the Met level in Arabidopsis plants overexpressing cytosolic MSRB7 [121]. Thereby, we can infer from these studies the occurrence of signaling crosstalk between cell sub-compartments resulting from impaired Met redox status in endoplasmic reticulum or cytosol and controlling nuclear gene expression and/or plastidial redox balance. In line with this conclusion, chloroplastic proteins are more prone to Met oxidation compared to other compartments in an Arabidopsis mutant deficient in *catalase 2* displaying increased H_2_O_2_ production in peroxisome [110].

#### 6.2.3. Antixoxidant Mechanisms

As reviewed in Section 3, modifying expression of *MSR* genes is often associated with changes in cell redox homeostasis. Thus, higher MetO levels are observed in Arabidopsis *MSR*-deficient mutants particularly upon environmental constraints [52,105,109]. Moreover, the *msra2* mutant exhibits increased levels of protein nitration and glycation [109]. These findings demonstrate the integration of MSRs in the cell antioxidant network and indicate that impairment of one specific MSR-based repair system leads to general disturbance in the protein redox balance. This could originate from altered activity of other antioxidant proteins due to change in their Met redox status. This hypothesis is corroborated by the facts that two catalase isoforms interact with MSRB [122] and that Arabidopsis plants over-expressing cytosolic *MSRBs* display upon oxidative stress modified activity levels of peroxidase and catalase, and most importantly strongly increased GST activity [51]. This could indicate protection of GSTs by MSRs. Consistently, one GST isoform exhibits reduced abundance in a mutant lacking MSRB3 [52], and three GSTs are more subject to Met oxidation in a *catalase 2* mutant than in WT upon high light [110]. Further, three GSTFs (2, 3 and 8) exhibit less oxidized Met residues in an Arabidopsis line overexpressing *MSRB7* exposed to MV [121].

GSTs form a complex family of enzymes detoxifying a broad range of molecules such as secondary metabolites and exogenous substrates that are referred as xenobiotics, and include herbicides [124,125,126]. Depending on their catalytic residues, GSTs catalyze glutathione conjugation, perform deglutathionylation or bind non-substrate ligands. The higher GST activity in Arabidopsis *MSRB*-overexpressing plants prompted Chan’s group [121] to investigate whether these enzymes are efficiently repaired by MSRs. Recombinant GSTFs 2, 3 and 8 interact with MSRB7 in BiFC, co-immunoprecipitation and yeast two-hybrid assays, and the activity of GSTFs 2 and 3 is restored in vitro by MSRB7 following oxidative treatment [121]. Moreover, plants overexpressing MSRB7 display a higher abundance of GSTFs 2 and 3 in oxidative stress conditions, revealing preserved protein stability possibly through the maintenance of Met redox status. In full agreement, MetO formation due to H_2_O_2_ treatment affects the activity of two other GSTs (GSTF9 and GSTT23) [110] and H_2_O_2_ leads to preferential oxidation of Met14 in GSTT23, that could alter GSH binding and/or catalytic activity of the enzyme [127]. Moreover, Met oxidation in GSTF9 results in increased flexibility in the H-site responsible of substrate binding and in lower enzyme activity towards hydrophobic substrates [128]. Taken collectively, these data give strong credence for a decisive role of plant MSRs in the redox maintenance of GSTs. Whether MSRs fulfil a similar function towards other antioxidant/detoxifying enzymes remains to be unveiled. Such a function seems very plausible, since in other organisms MSRs protect the activity of H_2_O_2_-scavenging enzymes [129].

#### 6.2.4. Signaling in Relation with Calmodulin

Ca^2+^ is a major signal carrier and messenger in eukaryotic cells, and different sensors recognize specific calcium signatures caused by exogenous stimuli. Among them, the calmodulin (CAM) calcium receptor is a key actor in animal and plant cells. CAM proteins display Met-rich pockets binding partners harboring non-polar peptide sequences [130]. The Met redox status in these pockets is thus critical for partner recognition due to the hydrophobic character of Met compared to MetO [131]. Accordingly, oxidized CAM and MSR exhibit high affinity and cooperative interaction in in vitro assays [132]. Reversible Met oxidation finely tunes CAM-dependent signaling and modulates interaction with CAM-binding proteins [133,134,135,136]. Besides, Met oxidation can also directly regulate CAM-partners as inferred from the increased activity of CAM-dependent kinase II in *msrA*-deficient mice [40]. All these findings clearly provide evidence for crosstalk between redox modification of Met and calcium-dependent signaling pathways.

Compared to other organisms, plants possess much more CAM and CAM-like proteins (ca. 50) that regulate numerous binding partners involved in developmental processes and stress responses [137,138]. So far, the knowledge regarding their regulation via Met oxidation remains scarce. Several proteins participating in Ca^2+^-dependent signaling are potential MSR substrates. Thus, among the 13 soybean drought-induced genes encoding Met-rich proteins, five code for CAM-related proteins [118]. In *G. soja*, the search of partners of a CAM-binding kinase led to the isolation of a MSRB isoform (B5a) [60]. The interaction was confirmed in BiFC assays and Arabidopsis lines overexpressing either MSRB5a or the CAM-binding kinase display enhanced tolerance to carbonate alkaline stress, suggesting that both fulfil a related physiological function. Intriguingly, the interaction takes place in the plasma membrane where the CAM-binding kinase is addressed, as well as MSRB5a when expressed without its plastidial transit peptide [60].

In plants, CAMs control many developmental processes including senescence. In litchi fruit pericarp, similar expression profiles were noticed for *CAM1* and three *MSR* genes during storage [61]. CAM1 physically interacts with two MSRA isoforms and can be repaired by MSRs after oxidation. Met oxidation in CAM1 does not alter its ability to bind two senescence-related transcription factors, but triggers their DNA-binding activity, revealing a possible role of MSRs in the control of the expression of senescence genes [61]. Very similar results were obtained in banana fruit [62]. These recent data reveal that like in animal cells the regulation of Met redox status in CAM-related proteins is likely a key step tuning their activity.

#### 6.2.5. Proteins Responsive to Stress

Finally, most proteomic strategies highlight the importance of the control of Met redox status in stress responsive proteins. Indeed, among the 121 and 213 Met-rich proteins in Arabidopsis and soybean, respectively, many respond to drought or high salt and participate in regulation of transcription, modification of proteins and transport of metals [118]. Consistently, among the proteins exhibiting MetO following treatment of Arabidopsis cell cultures with a cGMP analogue, proteins responsive to various stress conditions such as tubulin or aconitase, are substantially enriched [111]. In *MSRB7*-overexpressing Arabidopsis plants, several proteins involved in stress responses and signaling processes (annexin D1, tubulins, transducins) carry less MetO residues than in WT [121]. Finally, in proteins subject to Met oxidation in an Arabidopsis *catalase 2*-deficient mutant, those belonging to the gene ontology (GO) biological process “response to stress” are enriched compared to WT [110]. Among them, not only executors like oxidoreductases, but also regulators involved in signal transduction like methylene-blue-sensitive 1 and mitogen-activated protein kinases are found.

## 7. Conclusions

The knowledge about the physiological functions of plant MSRs has considerably evolved in recent years, and their participation in defense mechanisms against abiotic and biotic constraints is now well established (Figure 3). They can act in the preservation of proteins upon oxidative stress, but could also be targets of pathogens as proposed by Gao et al. [54]. These data could open up avenues for improving crop responses to environmental stress conditions. Moreover, another function related to the control of longevity was unveiled in seeds subjected to adverse conditions [68], which is in agreement with the data reported in other organisms. The *MSR* expression patterns observed in leaf and fruit as a function of age or maturation stage, respectively, in various plant species prompt us to propose that MSRs participate in the control of senescence and ageing processes in plant organs (Figure 3).

**Figure 4:**
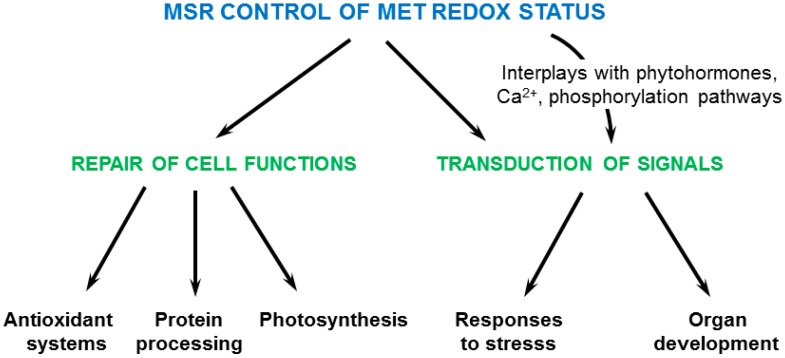
Presumed modes of action of plant MSRs. The proposed functions of MSRs in repair and preservation of antioxidant, protein processing, and photosynthetic systems and in signaling transduction pathways are based on the phenotype of plants modified for *MSR* expression and on the identity of their possible targets.

Most interestingly, the search of partners provided accurate information how plant MSRs could fulfill their functions upon environmental constraints (Figure 4). Indeed, in addition to a global and direct protective role against oxidative damage in proteins, for instance in photosynthetic structures, MSRs on one hand could maintain the Met redox status preferentially in stress responsive effectors, thereby preserving their activity as shown for GSTs and chaperones. On the other hand, they very likely play key signaling roles in relation with Ca^2+^-, hormone- and phosphorylation-dependent cascades as inferred from the identification of numerous MSR partners involved in these pathways [60,61,62,110,118]. The control of Met redox status by MSRs could be responsible for decoding ROS signatures and transmitting information in non-redox signaling pathways. Consistently, oxidation of Met538 in Arabidopsis nitrate reductase prevents in vivo phosphorylation of a nearby Ser residue, revealing the control of oxidative signals such as MetO formation on the capacity of kinase substrates to be phosphorylated due to modified recognition motif [139], MetO being more hydrophilic than Met. The hypothesis that Met oxidation participates in the control of protein phosphorylation is further supported by the fact that Met residues nearby phosphorylation sites are preferentially oxidized in vivo under stress conditions in human proteins [140]. These data indicate that MSRs are decisive components at the crosstalk of different transduction pathways within the complex signaling network (Figure 4).

In addition to reductase activity, plant MSRs might fulfill other biochemical functions, since some do not harbor any catalytic cysteine [32], and bacterial and animal MSRAs exhibit in vitro methionine oxidase activity towards both free and peptide-bound Met [141]. Further investigation is thus needed to decipher more precisely *in planta* the functions of each MSR isoform, notably by identifying using redox proteomics the MetO-containing proteins in various genetic backgrounds and environmental conditions. Another promising approach consists of determining the Met redox status in vivo using redox fluorescent sensors like in bacterial and mammalian cells [14]. This will help with monitoring the MetO level in various subcellular compartments and genetic backgrounds as a function of environmental stimuli.

## Figures and Tables

**Table 1:** Effects of various constraints and treatments on the expression of *methionine sulfoxide reductases* (*MSR*) genes in higher plants. The table was built from data gained on plants exposed to the mentioned constraints and treatments (in bold) either in vivo or in vitro. The cited reports aimed to investigate the expression of one or more *MSR* (*A* or *B*) genes at the transcript or protein levels. ↗, the expression of at least one *MSR* gene is up-regulated; ↘, the expression of at least one *MSR* gene is down-regulated; ↗ ↘, the expression of at least two *MSR* genes is modified (up or down).

Condition	Variation in *MSR* Expression. Species	References
Abiotic constraints		
High light	↗ *A. thaliana*	[76]
	↗ *S. cereale*	[67]
High light/low temperature	↗ *A. thaliana*	[50]
Low temperature	↗ *N. tabacum*, *S. lycopersicum*	[59,82]
	↗ *O. sativa*, *S. cereale*, *Z. mays*	[55,67,81]
Water deficit	↗ *G. max*, *N. tabacum*	[58,69]
High salt (NaCl)	↗ *A. halimus*, *A. thaliana*, *N. tabacum*, *S. lycopersicum*	[58,59,82,83,96]
	↗ *H. vulgare*, *O. sativa*, *Z. mays*	[48,55,84]
	↗ ↘ *G. max*	[69]
High carbonate	↗ *G. soja*	[60]
Cadmium	↗ ↘ *A. thaliana*, *B. juncea*	[78,79,80]
Biotic constraints		
Virus	↗ *A. thaliana*, *C. papaya*	[49,54]
Bacteria	↗ *A. thaliana*	[86]
	↘ *C. annuum*	[56]
Fungi	↗ ↘ *Populus × interamericana*	[50]
Parasite plants	↗ *A. thaliana*	[87]
Oxidative treatments		
Methyl viologen	↗ *A. thaliana*, *N. tabacum*, *S. lycopersicum*	[51,59,76,82]
	↗ *O. sativa*, *S. cereale*	[55,67]
Hydrogen peroxide	↗ ↘ *S. lycopersicum*	[82]
	↗ *A. thaliana*	[96]
Singlet oxygen	↗ *A. thaliana*	[97]
*S*-nitrosoglutathione	↗ *A. thaliana*	[98]
Copper excess	↗ *O. sativa*	[70]
Hormone treatments		
Abscisic acid	↗ ↘ *A. thaliana*, *G. max*, *N. tabacum*, *S. lycopersicum*	[58,59,69,82,96]
Jasmonic acid	↗ ↘ *A. thaliana*, *S. lycopersicum*	[82,96,100]
	↘ *C. annuum*	[56]
Salicylic acid	↗ ↘ *S. lycopersicum*	[82]
	↘ *C. annuum*	[56]
Ethylene	↗ *S. lycopersicum*	[82]
	↗ *M. acuminata*	[62]
